# Dysregulation of a lncRNA within the *TNFRSF10A* locus activates cell death pathways

**DOI:** 10.1038/s41420-023-01544-5

**Published:** 2023-07-13

**Authors:** Tadeusz J. Kaczynski, Nadine J. Husami, Elizabeth D. Au, Michael H. Farkas

**Affiliations:** 1 Research Service, VA Medical Center, Buffalo, NY USA; 2 Department of Ophthalmology, State University of New York at Buffalo, Buffalo, NY USA; 3 Department of Biochemistry, State University of New York at Buffalo, Buffalo, NY USA

**Keywords:** RNA, Gene regulation, Necroptosis

## Abstract

*TNFRSF10A (tumor necrosis factor receptor superfamily member 10A)* encodes a cell surface receptor protein involved in apoptotic, necroptotic, and inflammatory pathways. Dysregulation of *TNFRSF10A* has been implicated in sensitization to apoptosis and to the development of multiple diseases, yet little is known of the *AC100861.1* long noncoding RNA (lncRNA) that lies head-to-head with *TNFRSF10A*. Given its genomic positioning, we sought to investigate the function of *AC100861.1*, focusing on its potential relationship with *TNFRSF10A* and the role it may play in death receptor signaling. Using knockdown and overexpression strategies, we probed cell viability and examined transcript and protein-level changes in key genes involved in apoptosis, necroptosis, and inflammation. Decreased cell viability was observed upon *TNFRSF10A* overexpression, regardless of whether the cells were subjected to the chemical stressor tunicamycin. Similarly, overexpression of *AC100861.1* led to increased cell death, with a further increase observed under conditions of cellular stress. Knockdown of *TNFRSF10A* increased cell death only when the cells were stressed, and *AC100861.1* knockdown exhibited no effect on cell death. Neither knockdown nor overexpression of either of these genes greatly affected the expression of the other. Manipulating *AC100861.1*, however, led to marked changes in the expression of genes involved in necroptosis and inflammatory cell-signaling pathways. Additionally, RNA fluorescence in situ hybridization (RNA-FISH) revealed that the *AC100861.1* transcript is localized primarily to the cytoplasm. Together, these data suggest that *AC100861.1* may have a role in regulating necroptotic and inflammatory signaling pathways and that this function is separate from changes in *TNFRSF10A* expression. Given the importance of this genomic locus for cell survival, these data provide insight into the function of a poorly understood lncRNA with potential implications regarding disease pathology and treatment.

## Introduction

The *TNFRSF10A* genomic locus contains three genes: the protein-coding *tumor necrosis factor receptor superfamily member 10A* (*TNFSFR10A*, also known as *death receptor 4*, *DR4*, and *TNF-related apoptosis-inducing ligand receptor 1*, *TRAIL-R1*) and two lncRNA genes: TNFRSF10A antisense RNA 1 (TNFRSF10A-AS1) and *AC100861.1* (also known as *TNFRSF10A-divergent transcript, TNFRSF10A-DT*, and *LOC389641*). Little is known about *AC100861.1* and how it might operate within the context of its genomic locus.

*TNFSFR10A* encodes a cell surface receptor involved in apoptotic, necroptotic, and inflammatory cell-signaling pathways [1–5]. TNFRSF10A serves as a receptor for the cytokine TNF-related apoptosis-inducing ligand (TRAIL) in death receptor signaling pathways and has also been shown to be involved in the activation of NFκβ inflammatory signaling pathways [6]. In FAS-associated death domain protein (FADD)-dependent apoptotic pathways, TRAIL binds to TNFRSF10A, which activates the receptor by exposing its cytoplasmic death domain [7, 8]. The exposed death domain binds the adapter molecule FADD, which is required for caspase 8 (CASP8) and caspase 10 (CASP10) activation. Upon activation, CASP8 can then induce apoptosis through either direct cleavage of caspase 3 (CASP3) or via cleavage of BH3-interacting domain death agonist (BID) protein for translocation to the mitochondria to stimulate cytochrome C release [9]. When CASP8 is inhibited, death receptor signaling can promote the formation of the necrosome and subsequent progression toward necroptosis [10]. Under high levels of CASP3 activation, apoptosis occurs. However, low levels of CASP3 activation have been implicated in promoting survival through the activation of inflammatory pathways [11]. These pathways can also be influenced by other cell surface receptors related to TNFRSF10A. TRAIL can bind TNFRSF10B to transduce an apoptosis signal, while TNFRSF10C and TNFRSF10D are thought to act as antagonistic receptors that protect cells from TRAIL-induced apoptosis [12, 13].

Most lncRNAs, like *AC100861.1*, have not been functionally characterized, yet their general functional mechanisms are fairly well understood. Molecularly similar to messenger RNAs (mRNAs), lncRNAs are transcripts greater than 200 nucleotides in length that do not contain any large open reading frames and thus do not generally encode proteins [14, 15]. Although the collective function of most characterized lncRNAs is to regulate gene expression, the exact mechanism of action employed by a particular lncRNA is determined, in part, by its subcellular localization [16–18]. Nuclear lncRNAs typically regulate epigenetic modifications, transcription, and transcript splicing [18, 19]. Cytoplasmic lncRNAs usually participate in post-transcriptional regulation of gene expression via alteration of target transcript processing, stability, and/or translation [18, 20]. Long noncoding RNAs have also been implicated in altering miRNA-mediated mRNA destabilization and siRNA-mediated mRNA degradation, both of which can be enacted within either the nucleus or cytoplasm [21].

It is currently unknown which, if any, of the known lncRNA functional mechanisms are employed by *AC100861.1* or whether it has a function at all. However, due to its genomic positioning, *AC100861.1* has the distinct potential to regulate *TNFRSF10A* expression. The two genes lie in a head-to-head orientation with each other and share the same promoter region [22]. This genomic orientation of *AC100861.1* is indicative of a natural antisense transcript (NAT), a type of lncRNA transcribed from the opposite strand of its associated protein-coding gene that has been implicated in the negative regulation of the associated sense transcript [23]. Whether *AC100861.1* regulates *TNFRSF10A* expression as a NAT or possesses another function, the significance of this locus underscores the importance of determining the function of this lncRNA.

Dysregulation of the *TNFRSF10A* locus may contribute to a variety of diseases. *TNFRSF10A* has been associated with conditions such as inflammatory diseases, age-related macular degeneration (AMD), and cancer [24–27]. In some of these diseases, mutations in the *TNFRSF10A* coding region can lead to the production of a dysfunctional protein that overly inhibits apoptotic induction [26]. In other cases, disease pathogenesis has been linked to altered expression of *TNFRSF10A*, which has been shown to increase susceptibility to apoptosis [28, 29]. *AC100861.1*, though poorly understood, has been implicated in the tumor progression of pancreatic, lung, and colon cancers [30–32]. Additionally, since the age-related macular degeneration (AMD) risk single nucleotide polymorphism (SNP), rs13278062, resides within the first exon of *AC100861.1*, and the minor allele potentially leads to downregulation of this lncRNA, it is possible that *AC100861.1* dysregulation could contribute to AMD pathogenesis [33–35].

Although the importance of this locus is apparent, several key questions remain: (1) what is the function of *AC100861.1*, (2) what is the relationship between *AC100861.1* and *TNFRSF10A*, and (3) what are the consequences of *AC100861.1* and *TNFRSF10A* dysregulation? Here, we sought to answer these questions using overexpression and knockdown strategies, finding that *AC100861.1* appears to regulate necroptotic and inflammatory pathways independently of *TNFRSF10A*. Though the exact mechanism through which *AC100861.1* achieves this remains unclear, this study highlights a concrete function for this lncRNA and, in doing so, paves the way for future experimentation.

## Results

### Expression of AC100861.1 and TNFRSF10A are independent of one another

**Fig. 1:**
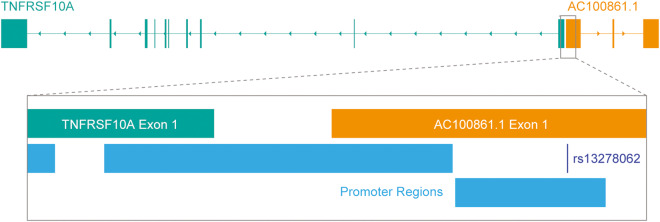
Schematic of the human *TNFRSF10A* genetic locus. The *AC100861.1* (orange) and *TNFRSF10A* (teal) genes possess a head-to-head orientation. The inset is a magnification of the promoter regions and the first exons of *AC100861.1* and *TNFRSF10A*. Promoter regions (light blue) are indicated. The rs13278062 SNP (dark blue) falls within the first exon of the *AC100861.1* gene.

As an initial step in characterizing the relationship between *AC100861.1* and *TNFRSF10A*, we sought to examine the *TNFRSF10A* locus using basic bioinformatic analyses. A pairwise sequence alignment of the two genes revealed that their primary sequences have approximately 40% homology [Supplementary Fig. S1]. We also noted the head-to-head genomic organization of the two genes with a shared promoter region between them [Fig. 1]. The sequence homology and gene orientation alluded to the potential for *AC100861.1* to regulate *TNFRSF10A* expression through antisense- or siRNA-mediated mechanisms [23, 36].

**Fig. 2:**
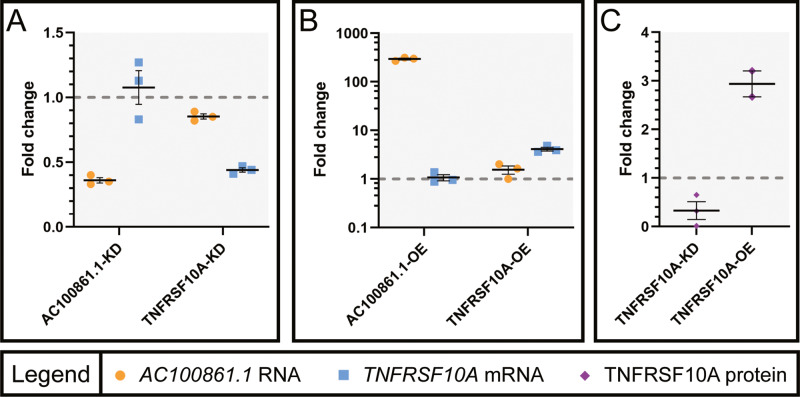
*AC100861.1* and *TNFRSF10A* transcripts levels do not affect the expression of each other in trans. Graphs indicate the changes in expression (compared to control) of *AC100861.1* and *TNFRSF10A* in ARPE-19 cells in response to siRNA-mediated knockdown (**A**) and vector overexpression (**B**), as measured by RT-qPCR. **C** Graph indicating the protein-level changes (compared to control) of TNFRSF10A in ARPE-19 cells in response to siRNA-mediated knockdown and vector overexpression, as measured by ELISA. The gray dotted line corresponds to fold change = 1. For TNFRSF10A-OE protein-level analysis in (**C**), *N* = 2. For all other samples, *N* = 3. Error bars indicate the standard error of the mean.

To investigate the relationship between transcript levels of *AC100861.1* and *TNFRSF10A*, the expression of each gene was manipulated in ARPE-19 cells via siRNA-mediated knockdown or vector-mediated overexpression. Expression levels were measured using RT-qPCR. Cells transfected with siRNA targeting *AC100861.1* (hereafter referred to as AC100861.1-KD) demonstrated a decreased expression of *AC100861.1* by approximately 64% (fold change (FC) = 0.36 ± 0.02), relative to non-transfected control cells [Fig. 2A]. Similarly, upon transfection with siRNA targeting *TNFRSF10A* (hereafter referred to as TNFRSF10A-KD), we observed decreased expression of *TNFRSF10A* by approximately 56% (FC = 0.44 ± 0.017), relative to non-transfected control cells [Fig. 2A]. This reduction in *TNFRSF10A* mRNA was accompanied by an approximate 67% (FC = 0.33 ± 0.18) reduction in TNFRSF10A protein [Fig. 2C]. Neither knockdown of *AC100861.1* nor the knockdown of *TNFRSF10A* greatly affected the expression of the other gene [Fig. 2A].

We then examined how overexpression of either *AC100861.1* or *TNFRSF10A* affected the expression of the other. Overexpression of *AC100861.1* (hereafter referred to as AC100861.1-OE) exhibited an approximate 300-fold increase in expression relative to non-transfected control cells (FC = 295.71 ± 12.96), yet the *TNFRSF10A* levels, relative to control, were not markedly affected (FC = 1.07 ± 0.0.16) [Fig. 2B]. Similarly, overexpression of *TNFRSF10A* (hereafter referred to as TNFRSF10A-OE) showed increased expression relative to control (FC = 4.12 ± 0.36), was accompanied by a similar increase in protein levels as measured by ELISA (FC = 2.94 ± 0.27), but did not substantially alter the expression of *AC100861.1* (FC = 1.55 ± 0.30) [Fig. 2B, C]. These results suggest that *AC100861.1* and *TNFRSF10A* do not regulate the expression of each other in trans.

### Dysregulation of AC100861.1 and TNFRSF10A increases susceptibility to cell death

Given the known functions of *TNFRSF10A*, we aimed to explore whether and in what ways dysregulation of the *TNFRSF10A* locus influences signaling pathways involved in cell death and survival. To determine whether altering the expression of the genes from this locus influences cell viability, we conducted Terminal Deoxynucleotidyl Transferase dUTP Nick End Labeling (TUNEL) assays. ARPE-19 cells were transfected as above (to overexpress or knockdown *AC100861.1* or *TNFRSF10A*), treated with either tunicamycin (Tm, to induce cell stress) or with dimethyl sulfoxide (DMSO, as vehicle control), and probed for the extent of cell death using TUNEL staining and flow cytometry.

**Fig. 3:**
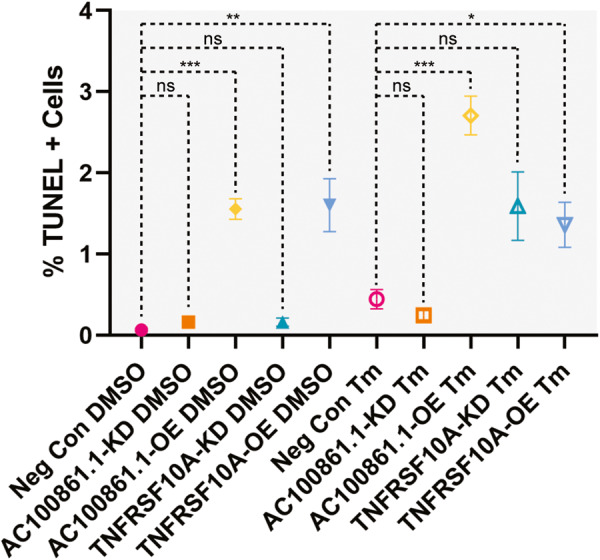
Dysregulation of *AC100861.1* and *TNFRSF10A* leads to increased cell death. The graph displays the percentage of TUNEL-positive cells from ARPE-19 cells transfected with: transfection reagent only (neg con, magenta circles), siRNA targeting *AC100861.1* (AC100861.1-KD, orange squares), *AC100861.1* overexpression vector (AC100861.1-OE, yellow diamonds), siRNA targeting *TNFRSF10A* (TNFRSF10A-KD, teal upward pointing triangles), and *TNFRSF10A* overexpression vector (TNFRSF10A-OE, blue downward pointing triangles). Samples were treated with either DMSO (filled shapes) or Tm (hollow shapes). For neg con samples, *N* = 10. For all other samples, *N* = 5. Error bars indicate the standard error of the mean. Black dotted lines indicate comparisons using unpaired, two-tailed *t*-tests with Welch’s correction with significance levels as shown (ns = not significant, * = *p* ≤ 0.05, ** = *p* ≤ 0.01, *** = *p* ≤ 0.001).

Within non-transfected control cells (neg con), treated with either DMSO or Tm, we found few TUNEL-positive cells, indicating low basal levels of programmed cell death (0.07% ± 0.02% and 0.44% ± 0.12% TUNEL-positive cells in DMSO- and Tm-treated samples, respectively) [Fig. 3]. AC100861.1-KD samples did not demonstrate significant cell death rates, as indicated by TUNEL staining (0.16% ± 0.06% and 0.25% ± 0.06% TUNEL-positive cells in DMSO- and Tm-treated samples, respectively) [Fig. 3]. On the other hand, in AC100861.1-OE samples, there was a significant increase in the percentage of TUNEL-positive cells in both the DMSO- and Tm-treated samples (1.55% ± 0.13% and 2.71% ± 0.24% TUNEL-positive cells in DMSO- and Tm-treated samples, respectively) [Fig. 3], with a significantly higher percentage observed in the Tm-treated samples compared to DMSO-treated samples (*p* ≤ 0.01). While neither the DMSO-treated nor the Tm-treated TNFRSF10A-KD samples displayed cell death rates significantly different from the non-transfected controls (0.16% 0.05% and 1.59% ± 0.42% TUNEL-positive cells, respectively) [Fig. 3], the Tm-treated TNFRSF10A-KD samples possessed levels of TUNEL-positive cells that were significantly elevated above those of the DMSO-treated TNFRSF10A-KD samples (*p* ≤ 0.05). Interestingly, in both DMSO- and Tm-treated TNFRSF10A-OE samples, we observed significant increases in the percentages of TUNEL-positive cells compared to non-transfected controls (1.60% ± 0.33% and 1.36% ± 0.28% TUNEL-positive cells in DMSO and Tm-treated samples, respectively) [Fig. 3]. Together, these data indicate that both overexpression of *AC100861.1* and dysregulation of *TNFRSF10A* lead to decreased cell viability, particularly under conditions of cellular stress.

### Altered expression of AC100861.1 increases expression of necroptotic pathway components

**Fig. 4:**
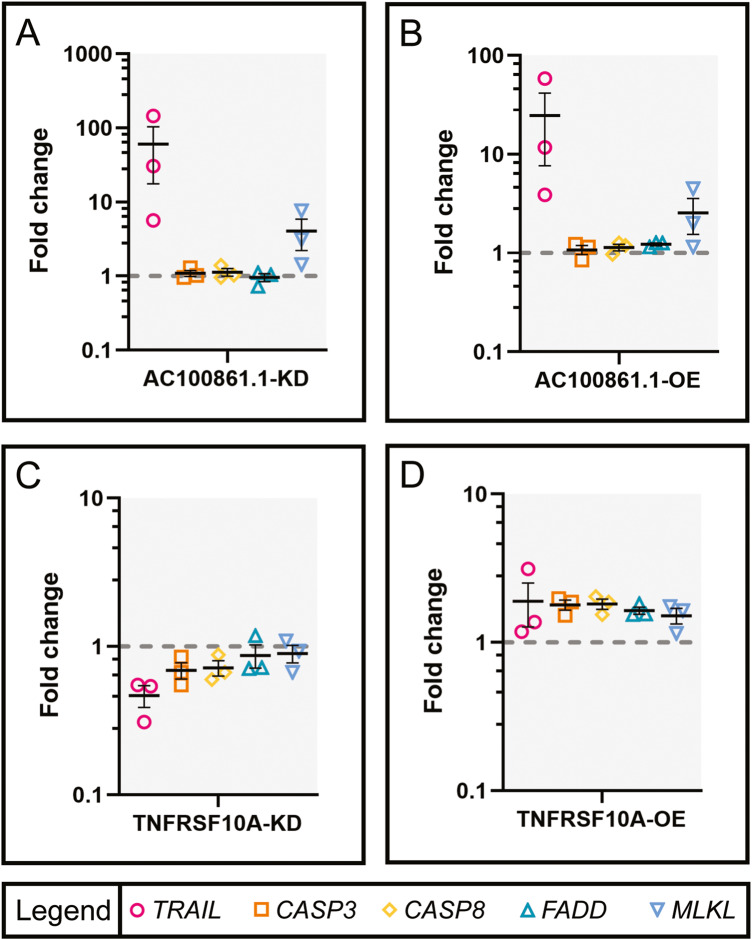
Components of cell death signaling pathways are affected by AC100861.1 and TNFRSF10A dysregulation. Graphs indicate the expression level changes (compared to control) in ARPE-19 cells transfected with siRNA targeting *AC100861.1* (**A**), *AC100861.1* overexpression vector (**B**), siRNA targeting *TNFRSF10A* (**C**), and *TNFRSF10A* overexpression vector (**D**). Expression levels of *TRAIL*, *CASP3*, *CASP8*, *FADD*, and *MLKL* were measured by RT-qPCR. The gray dotted line corresponds to fold change = 1. For all samples, *N* = 3. Error bars indicate the standard error of the mean.

Having determined that dysregulation of both *AC100861.1* and *TNFRSF10A* results in increased cell death, we next wanted to examine how the expression of apoptotic and necroptotic pathway components might change under these conditions. To this end, we performed RT-qPCR analyses using primers targeting *TRAIL*, *CASP3*, *CASP8*, *FADD*, and the necroptotic signaling component mixed lineage kinase domain-like protein (*MLKL*) [Supplementary Table S2]. Expression of *TRAIL* was dramatically upregulated in both AC100861.1-KD and AC100861.1-OE samples (FC = 60.51 ± 42.97 and 24.61 ± 16.97, respectively) [Fig. 4A, B]. Curiously, this upregulation of *TRAIL* in response to dysregulated *AC100861.1* was highly variable between biological replicates, ranging from a 5.64 fold to a 145.23 fold increase in the AC100861.1-KD samples and a 3.88 fold to a 58.25 fold increase in the AC100861.1-OE samples [Fig. 4A, B]. *TRAIL* expression under *TNFRSF10A* dysregulation was less dramatically altered, showing a moderate decrease in TNFRSF10A-KD samples and a minimal increase in TNFRSF10A-OE samples (FC = 0.47 ± 0.08 and 1.89 ± 0.62, respectively) [Fig. 4C, D]. *CASP3*, *CASP8*, and *FADD* expression levels were virtually unchanged in AC100861.1-KD (FC = 1.09 ± 0.10, 1.13 ± 0.14, and 0.96 ± 0.12, respectively) and AC100861.1-OE (FC = 1.08 ± 0.12, 1.14 ± 0.09, and 1.23 ± 0.04, respectively) samples [Fig. 4A, B]. Similarly, expression of *CASP3*, *CASP8*, and *FADD* was only slightly decreased in TNFRSF10A-KD samples (FC = 0.69 ± 0.09, 0.72 ± 0.08, and 0.87 ± 0.16, respectively) and only marginally increased in TNFRSF10A-OE samples (FC = 1.79 ± 0.14, 1.82 ± 0.15, and 1.64 ± 0.09, respectively) [Fig. 4C, D]. *MLKL* expression was significantly increased in both AC100861.1-KD and AC100861.1-OE samples (FC = 4.05 ± 1.84 and 2.55 ± 1.00, respectively) [Fig. 4A, B]. TNFRSF10A-KD and TNFRSF10A-OE samples, however, displayed minimal changes in *MLKL* expression (FC = 0.90 ± 0.12 and 1.51 ± 0.18, respectively) [Fig. 4C, D].

**Fig. 5:**
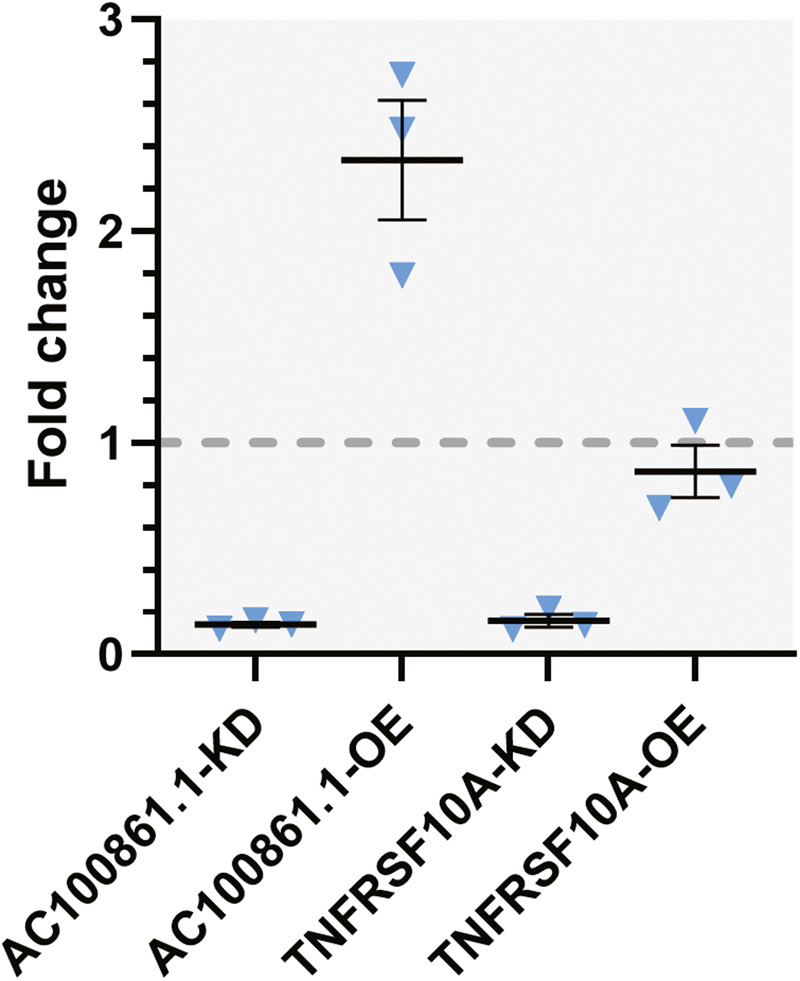
Phosphorylated MLKL levels change in response to AC100861.1 and TNFRSF10A dysregulation. Graph displaying the fold change of phosphorylated MLKL (phos-MLKL) protein levels in the AC100861.1-KD, AC100861.1-OE, TNFRSF10A-KD, and TNFRSF10A-OE samples compared to control samples. The gray dotted line corresponds to fold change = 1. For all samples, *N* = 3. Error bars indicate the standard error of the mean.

We next examined whether dysregulated *AC100861.1* or *TNFRSF10A* affected the levels of cleaved CASP3 and phosphorylated MLKL (phos-MLKL) proteins, which are critical effector molecules for carrying out apoptosis and necroptosis, respectively. Cleaved CASP3 was not detectable within our samples as determined by ELISA. On the other hand, phos-MLKL, as detected by ELISA, was downregulated in the AC100861.1-KD and TNFRSF10A-KD samples (FC = 0.14 ± 0.01 and 0.16 ± 0.03, respectively), upregulated in the AC100861.1-OE samples (FC = 2.34 ± 0.28), and unchanged in the TNFRSF10A-OE samples (FC = 0.86 ± 0.12) [Fig. 5].

**Fig. 6:**
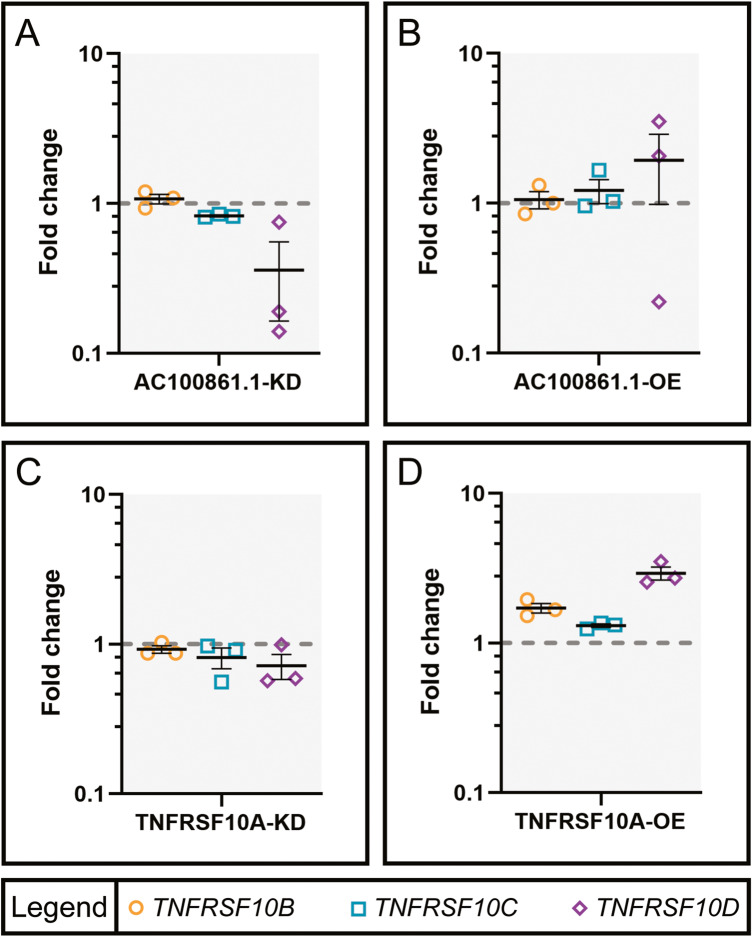
Expression of TRAIL receptors shifts after AC100861.1 and TNFRSF10A dysregulation. Graphs indicate the expression level changes (compared to control) in ARPE-19 cells transfected with siRNA targeting *AC100861.1* (**A**), *AC100861.1* overexpression vector (**B**), siRNA targeting *TNFRSF10A* (**C**), and *TNFRSF10A* overexpression vector (**D**). Expression levels of TNFRSF10B, TNFRSF10C, and TNFRSF10D were measured by RT-qPCR. The gray dotted line corresponds to fold change = 1. For all samples, *N* = 3. Error bars indicate the standard error of the mean.

Considering the changes in TRAIL expression in response to *AC100861.1* and *TNFRSF10A* dysregulation, we investigated whether the expression levels of TRAIL receptor genes, *TNFRSF10B*, *TNFRSF10C*, and *TNFRSF10D*, were also altered upon manipulation of *AC100861.1* or *TNFRSF10A*. We performed RT-qPCR analysis, which revealed that the expression of *TNFRSF10B* and *TNFRSF10C* were virtually unchanged in AC100861.1-KD (FC = 1.07 ± 0.08 and 0.83 ± 0.01, respectively), AC100861.1-OE (FC = 1.06 ± 0.14 and 1.22 ± 0.22, respectively), TNFRSF10A-KD (FC = 0.92 ± 0.05 and 0.81 ± 0.81, respectively), and TNFRSF10A-OE (FC = 1.71 ± 0.13 and 1.31 ± 0.04, respectively) samples [Fig. 6]. *TNFRSF10D* expression, on the other hand, was reduced in AC100861.1-KD and TNFRSF10A-KD samples (FC = 0.36 ± 0.20 and 0.72 ± 0.14, respectively) but increased in AC100861.1-OE and TNFRSF10A-OE samples (FC = 1.94 ± 0.96 and 2.93 ± 0.29, respectively) [Fig. 6].

### AC100861.1 and TNFRSF10A dysregulation alters the expression of inflammation pathway genes

**Fig. 7:**
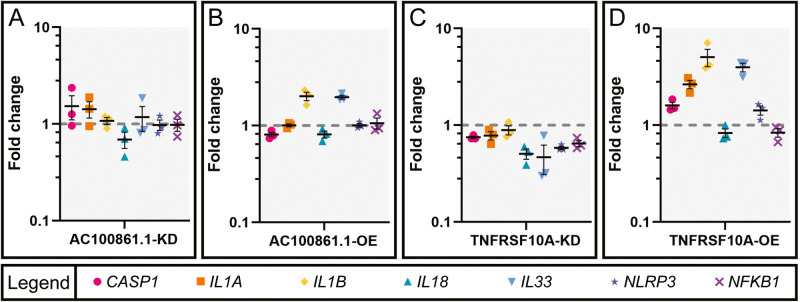
Components of inflammatory pathways are affected by *AC100861.1* and *TNFRSF10A* dysregulation. Graphs indicate the expression level changes (compared to control) in ARPE-19 cells transfected with siRNA targeting *AC100861.1* (**A**), *AC100861.1* overexpression vector (**B**), siRNA targeting *TNFRSF10A* (**C**), and *TNFRSF10A* overexpression vector (**D**). Expression levels of *CASP1*, *IL1A*, *IL1B*, *IL18*, *IL33*, *NLRP3*, and *NFKB1* were measured by RT-qPCR. The gray dotted line corresponds to fold change = 1. For all samples, *N* = 3. Error bars indicate the standard error of the mean.

Due to the effects of *AC100861.1* and *TNFRSF10A* dysregulation on cell viability, and given the interplay between programmed cell death and inflammation, we next looked at whether knockdown or overexpression of *AC100861.1* and *TNFRSF10A* affects the expression levels of genes involved in inflammatory signaling pathways. We quantified the expression levels of multiple inflammation pathway components using RT-qPCR with primers targeting: *caspase1* (*CASP1*), *interleukin 1 alpha* (*IL1A*), *interleukin 1 beta* (*IL1B*), *interleukin 18 (IL18), interleukin 33 (IL33), NLR family pyrin domain containing 3 (NLRP3)*, and *nuclear factor kappa B subunit 1* (*NFKB1*) [Supplementary Table S2]. *CASP1* expression was minimally affected by *AC100861.1* and *TNFRSF10A* dysregulation, with slight decreases observed in AC100861.1-OE (FC = 0.81 ± 0.04) and TNFRSF10A-KD (FC = 0.75 ± 0.02) samples and slight increases observed in AC100861.1-KD (FC = 1.53 ± 0.43) and TNFRSF10A-OE (FC = 1.60 ± 0.13) samples [Fig. 7]. Expression of *IL1A* was largely unaffected in AC100861.1-KD, AC100861.1-OE, and TNFRSF10A-KD samples (FC = 1.42 ± 0.27, 1.01 ± 0.04, and 0.78 ± 0.08, respectively), but it was markedly increased in TNFRSF10A-OE samples (FC = 2.65 ± 0.26) [Fig. 7]. Interestingly, *IL1B* was found to be unchanged in AC100861.1-KD and TNFRSF10A-KD samples (FC = 1.08 ± 0.09 and 0.89 ± 0.10, respectively) but upregulated in AC100861.1-OE and TNFRSF10A-OE samples (FC = 2.01 ± 0.20 and 5.06 ± 1.02, respectively) [Fig. 7]. *IL18* was mildly affected by AC100861.1 and TNFRSF10A dysregulation, being slightly downregulated in each of the AC100861.1-KD, AC100861.1-OE, TNFRSF10A-KD, and TNFRSF10A-OE samples (FC = 0.69 ± 0.13, 0.81 ± 0.07, 0.51 ± 0.06, and 0.83 ± 0.09, respectively) [Fig. 7]. *IL33* expression was decreased in TNFRSF10A-KD samples (FC = 0.47 ± 0.16), unchanged in AC100861.1-KD samples (FC = 1.18 ± 0.34), and increased in AC100861.1-OE and TNFRSF10A-OE samples (FC = 1.97 ± 0.09 and 3.97 ± 0.41, respectively) [Fig. 7]. *NLRP3* and *NFKB1* expression levels were decreased in TNFRSF10A-KD samples (FC = 0.58 ± 0.02 and 0.65 ± 0.05, respectively), and were minimally altered in AC100861.1-KD samples (FC = 0.98 ± 0.12 and 0.98 ± 0.14, respectively), AC100861.1-OE samples (FC = 1.01 ± 0.04 and 1.06 ± 0.14, respectively), and TNFRSF10A-OE samples (FC = 1.42 ± 0.15 and 0.84 ± 0.09, respectively) [Fig. 7]. Taken together, these data suggest that dysregulated levels of *AC100861.1* and *TNFRSF10A* influence the expression of IL1B and IL33 but have relatively little effect on other inflammation pathway genes.

### AC100861.1 localizes to the cytoplasm in ARPE-19 cells

**Fig. 8:**
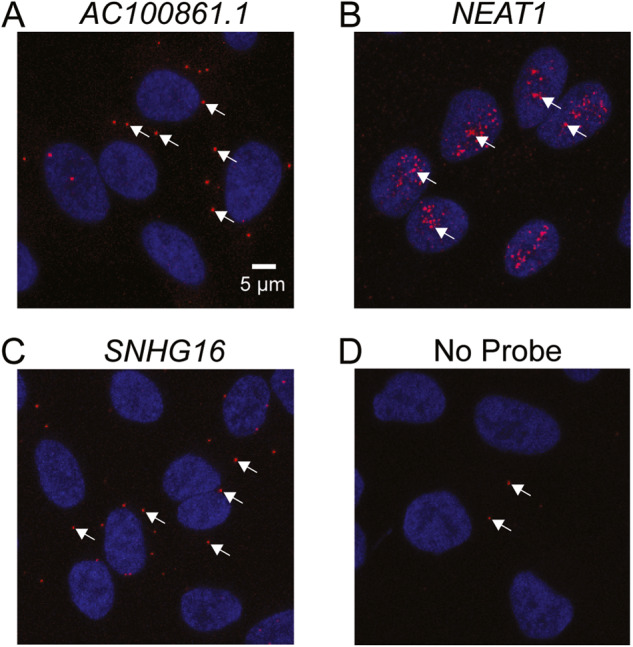
*AC100861.1* localizes to the cytoplasm. RNA fluorescent in situ hybridization (RNA-FISH) was performed on ARPE-19 cells using targeting probes against *AC100861.1* (**A**), the nuclear lncRNA *NEAT1* (**B**), and the cytoplasmic lncRNA *SNHG16* (**C**). **D** Minimal background signal is present in ARPE-19 cells subjected to RNA-FISH without the use of targeting probes against any RNA but with the use of the fluorescent label. Red dots indicate the fluorescent label conjugated to the targeted RNA, and arrows highlight examples of labeling from each image. Nuclei were counterstained with Hoechst (blue).

In order to gain insight into its functional mechanisms, we set out to determine the subcellular localization of the *AC100861.1* lncRNA. We utilized RNA-FISH to map the localization of the transcript within ARPE-19 cells and discovered that *AC100861.1* primarily localized to the cytoplasm [Fig. 8]. This localization was further supported by data obtained from previous work in our laboratory mapping the subcellular localization of the lncRNA transcriptome within iPSC-derived retinal pigment epithelium (iPSC-RPE) [37]. Using those data sets, we found that within the iPSC-RPE, *AC100861.1* transcripts localized to the cytoplasm, which helps to corroborate our RNA-FISH localization observations [Fig. 8].

## Discussion

In the current study, we investigated two genes within the *TNFRSF10A* locus, *AC100861.1* and *TNFRSF10A*. We sought to uncover: (1) the relationship between the expression of these two genes, (2) whether and to what extent their dysregulation impacts cellular viability and inflammation, and (3) insights into the function and mechanism of the previously uncharacterized *AC100861.1* lncRNA.

We found that, while *AC100861.1* possesses homology to *TNFRSF10A* and the genomic orientation reminiscent of a NAT, *AC100861.1* does not regulate the expression of *TNFRSF10A* in trans [Fig. 2]. Similarly, *TNFRSF10A* knockdown and overexpression failed to affect *AC100861.1* expression in trans [Fig. 2]. These data exclude the trans-regulation of *TNFRSF10A* as a possible functional mechanism of the *AC100861.1* lncRNA. It is important to note that these data do not expound on the possibility that these genes may regulate one another in *cis*.

Our data also revealed that dysregulation of both *AC100861.1* and *TNFRSF10A* leads to increased cell death. Regarding *TNFRSF10A*, we observed that both knockdown (when coupled with Tm-induced cellular stress) and overexpression (regardless of treatment condition) elicited TUNEL-positive staining above the corresponding controls [Fig. 3]. Prior research by Zhang et al. [38] and by Li et al. [25] showed that endoplasmic reticulum (ER) stress (such as that caused by Tm exposure [39–41]) led to sensitization to TRAIL, increased *TNFRSF10A* expression, and apoptosis. However, the minimal cell death we observed in non-transfected cells treated with Tm evinces a more complicated story, reflecting the different cell types and Tm concentrations used in our studies. Indeed, the concentration of Tm used in the current study was chosen to lie within the sub-lethal range based on our cell viability analysis conducted in ARPE-19 cells [Supplementary Fig. S2]. Considering that we observed that *TNFRSF10A* knockdown induced cell death only in conjunction with Tm treatment, it appears that diminished death receptor signaling through *TNFRSF10A* sensitizes cells to further insults and death. Overall, these data corroborate findings from multiple studies indicating that tightly controlled *TNFRSF10A* expression is critical for cell health [28, 29]. Regarding *AC100861.1*, knockdown had no effect on cell viability; however, overexpression led to an increase in cell death, which was further exacerbated by Tm-induced cellular stress [Fig. 3]. These experiments, which are the first to probe *AC100861.1* function in non-cancerous cells, allude to a property of *AC100861.1* that sensitizes cells towards cell death pathways. We acknowledge that the changes in TUNEL-positive cells observed in our experiments are modest, and while it might be tempting to discount such changes as inconsequential, it is important to keep in mind that such small changes can have a large impact over time, which is particularly relevant when considering the long time course of diseases impacted by dysregulation of the *TNFRSF10A* locus.

When we examined the cell death pathway changes in the AC100861.1-OE and AC100861.1-KD samples, we found evidence for *AC100861.1* involvement in necroptotic signaling. The sharp increase of *TRAIL* expression in AC100861.1-KD and AC100861.1-OE samples was indicative of death receptor signaling [Fig. 4]. Despite this increase in *TRAIL* expression, *AC100861.1* dysregulation yielded virtually no change in the expression levels of *CASP3*, *CASP8*, and *FADD* [Fig. 4]. These results suggest a lack of apoptotic signaling through the death receptor pathway. However, in the AC100861.1-KD and AC100861.1-OE samples, *MLKL*, which is a critical necroptotic pathway component that can be activated through death receptor signaling [42, 43], was upregulated at the transcript level [Fig. 4]. Interestingly, the active, phosphorylated MLKL protein was found to be upregulated in the AC100861.1-OE samples but downregulated in the AC100861.1-KD samples [Fig. 6]. This, along with the increase in cell death associated with the AC100861.1-OE samples [Fig. 3], suggests a connection between *AC100861.1* upregulation and increased necroptosis. Although AC100861.1-KD samples displayed increased expression of *TRAIL* and *MLKL* transcripts, they possessed reduced levels of phos-MLKL along with minimal levels of cell death [Fig. 3], indicating that the knockdown of *AC100861.1* does not appear to promote necroptosis. Collectively, these data point to the *AC100861.1* lncRNA as potentially cytotoxic and/or a possible regulator of necroptotic signaling.

Cell death pathways are also affected by the dysregulation of *TNFRSF10A*. Considering that TNFRSF10A-OE samples demonstrated decreased cell viability, an increase in the expression of *CASP3*, *CASP8*, and *FADD*, and virtually no change in phos-MLKL [Figs. 3, 4 and 6], our data support the notion that, unlike *AC100861.1*, upregulated *TNFRSF10A* leads to apoptosis rather than necroptosis. Other studies have come to similar conclusions, finding increased sensitivity to TRAIL-induced apoptotic signaling in response to upregulated *TNFRSF10A* [44–47]. On the other hand, we observed that the downregulation of TNFRSF10A led to decreased *CASP3*, *CASP8*, *FADD*, and *MLKL* expression, along with a decrease in phos-MLKL levels [Figs. 4 and 6]. These data indicate that decreased levels of *TNFRSF10A* dysregulate signaling pathways in a distinctly different manner from *TNFRSF10A* upregulation. A previous study by Mori et al. [28] found that the downregulation of *TNFRSF10A* caused an increase in cell death by decreasing protein kinase C-alpha (*PKCA*) expression levels, leading to the inactivation of the protein kinase C pathway. We did not observe such marked changes in *PKCA* levels in our samples, perhaps reflecting cell type differences [Supplementary Fig. S3]. It should also be noted that our inability to detect cleaved CASP3 in our samples is likely to reflect the relatively low levels of cell death in our samples and the detection limits of our ELISA analyses rather than a complete lack of apoptotic signaling.

Dysregulated *AC100861.1* and *TNFRSF10A* proved to have only minor effects on the expression of other TRAIL receptors. While the expression levels of *TNFRSF10B* and *TNFRSF10C* were almost wholly unchanged in any sample set, *TNFRSF10D* expression was found to be upregulated in the AC100861.1-OE and TNFRSF10A-OE samples and downregulated in the AC100861.1-KD and TNFRSF10A-KD samples [Fig. 5]. The exact causes and consequences of these expression changes are unclear, but the *TNFRSF10D* upregulation in the AC100861.1-OE and TNFRSF10A-OE samples hints at an attempted survival mechanism whereby the cells would avoid TRAIL-induced cell death through an increase in the production of antagonistic TRAIL receptors.

Along with alterations in the cell death signaling pathways, we found changes in the expression of inflammation signaling components resulting from *AC100861.1* and *TNFRSF10A* dysregulation. Largely, these changes were observed in the AC100861.1-OE and TNFRSF10A-OE samples, with IL1B and IL33 being upregulated in both sample sets [Fig. 6]. Both IL1B and IL33 are involved in macrophage recruitment to damaged cells and tissues [48]. IL33 is among a class of molecules known as damage-associated molecular patterns (DAMPs) that can be released following apoptosis and necroptosis, and the release of such DAMPs can instigate an inflammatory response, highlighting one aspect of the crosstalk between programmed cell death and inflammation [49, 50]. Although the complex interplay between inflammation, apoptosis, and necroptosis can be difficult to tease apart, based on our data, it appears likely that dysregulation of *AC100861.1* and *TNFRSF10A* trigger cascades of intracellular events that lead to programmed cell death, which then brings about DAMP release and inflammation signaling. More studies are needed to determine the veracity of this hypothesis.

Although somewhat disparate from the other analyses of this study, our investigation into the subcellular localization of the *AC100861.1* lncRNA is highly relevant to uncovering its function. The potential functions of a lncRNA are vast, and identifying localization can help narrow down the possibilities. Using RNA-FISH, we found *AC100861.1* to localize primarily to the cytoplasm [Fig. 8]. In the cytoplasm, a lncRNA can function to: (1) regulate mRNA turnover and mRNA translation, (2) regulate protein turnover, (3) act as a decoy for RNA binding proteins or microRNAs, and (4) modulate signaling pathways [20]. Given our observations that its dysregulation leads to altered gene expression and increased cell death, *AC100861.1* could theoretically operate in any of these four cytoplasmic lncRNA roles. Thus, while we are closer to identifying a functional mechanism for *AC100861.1*, RNA-protein pulldowns and other experimentation will be required to expand the groundwork laid out in this study.

It is important to note that the differences we observe in mRNA levels do not necessarily correspond to the same changes in protein levels of the respective genes. A wide variety of factors, either global or transcript specific, can influence translation, leading to discrepancies between mRNA and protein levels [51]. Indeed, the differences in the *TNFRSF10A* mRNA and TNFRSF10A protein levels in both the TNFRSF10A-KD and the TNFRSF10A-OE samples, while usually similar in direction and degree, are not perfect reflections of one another [Fig. 2]. However, by using transcript level changes as proxies for how *AC100861.1* and *TNFRSF10A* dysregulation is likely to affect the abundance of the protein components of the cell death and inflammation pathways, we gained a broad overview of the system. Then, using ELISA experimentation to quantify TNFRSF10A and key effector proteins involved in apoptosis (cleaved CASP3) and necroptosis (phos-MLKL), we narrowed our focus and identified the pathways affected by *AC100861.1* and *TNFRSF10A* dysregulation.

Overall, these data contribute to a more comprehensive understanding of the *TNFRSF10A* locus. While the positioning of *TNFRSF10A* within the death receptor signaling cascade has been previously described, here we build upon that knowledge and investigate the downstream consequences of *TNFRSF10A* dysregulation. This study definitively identifies multiple apoptotic and inflammatory genes with altered expression resulting from changes in *TNFRSF10A* expression, highlighting avenues through which changes in *TNFRSF10A* expression could contribute to cell dysfunction and disease. Moreover, our study is the first to conduct an in-depth investigation of the *AC100861.1* lncRNA. Though *AC100861.1* has features resembling a NAT, ultimately, our data indicate that *AC100861.1* does not function as a NAT to regulate *TNFRSF10A* transcription. Rather the two genes appear to function independently at the transcriptional level. Furthermore, we provide evidence to suggest that *AC100861.1* dysregulation sensitizes cells to necroptosis and local inflammation, which is likely the result of the disrupted expression of key nodes shared between the cell death and inflammation pathways. Crosstalk between these pathways muddles the identification of the point of *AC100861.1* involvement, but subcellular localization suggests that *AC100861.1* interacts with binding partners in the cytoplasm [Fig. 8]. Potential mechanisms of a cytoplasmic lncRNA include the regulation of mRNA translation, mRNA degradation, protein activity, and protein turnover through interactions with mRNAs, miRNAs, and RNA-binding proteins [20]. Additional studies of *AC100861.1* are needed to identify such binding partners, to illuminate what function the transcript might provide within a healthy cell, and to better understand how its dysregulation might contribute to disease.

### Limitations

It is worth noting that the ARPE-19 cell line used in this study has recently been criticized as a poor model for native human retinal pigment epithelium (RPE) [52]. The authors describe the limitations of ARPE-19 cells, citing several chromosomal abnormalities and transcriptomic differences between the cell line and native human RPE. We agree that ARPE-19 cells are not suitable in most circumstances as a model for human RPE. Indeed, our study is a cell biology study whose merits do not rely upon the similarities between ARPE-19 cells and human RPE. Although an exploration of *AC100861.1* and *TNFRSF10A* dysregulation within the RPE would prove relevant and worthwhile with regard to our understanding of AMD disease pathology, this study does not purport to provide such insights. Instead, the data presented here are meant to provide a foundation for future research conducted in contexts that are more relevant to specific cells and disease states.

## Materials and methods

### Culturing of cell lines

All reagents were purchased from Invitrogen (Carlsbad, CA, USA) unless noted otherwise. ARPE-19 cells (line APRE-19, ATCC [Manassas, VA, USA], Cat#: CRL-2302) were cultured in 49% Advanced DMEM (Fisher Scientific [Waltham, MA, USA], Cat #: 12-491-015), 49% F-12 (Fisher Scientific, Cat #: MT10080CV), and 2% FBS (ATCC, Cat#: 30-2020). For transfection experimentation, cells were transfected at approximately 70% confluency. For RNA-FISH experimentation, cells were utilized 24–48 h after reaching confluence. The ARPE-19 cells were tested and found to be negative for mycoplasma contamination. Cell line authentication was not performed.

### Cell transfection

Plasmids were constructed to express AC100861.1 or TNFRSF10A under the control of CMV promoters using standard cloning procedures. ARPE-19 cells were transfected with the Invitrogen Lipofectamine 3000 Transfection Reagent (Fisher Scientific, Cat #: L3000008) alone (non-transfected control) or with one of the following: an AC100861.1 expression vector, a TNFRSF10A expression vector, a control pCAGIG GFP expression vector (Addgene [Watertown, MA, USA], Cat #: 11159), an siRNA targeting AC100861.1 (Integrated DNA Technologies [Coralville, IA, USA], Cat#: CD.Ri.226263.13.1), an siRNA targeting TNFRSF10A (Integrated DNA Technologies, Cat#: hs.Ri.TNFRSF10A.13.1), or the TYE 563 Transfection Control DsiRNA (Integrated DNA Technologies, Cat#: 51-01-20-19). Sequences of the siRNAs targeting AC100861.1 and TNFRSF10A can be found in Supplementary Table S1. Transfections were carried out according to the manufacturer’s protocol.

### Cell stressor treatment

For TUNEL assay experimentation, tunicamycin (Tm, Abcam [Cambridge, United Kingdom], Cat#: ab120296) and dimethyl sulfoxide (DMSO, Krackeler [Albany, NY, USA], Scientific, Cat#: 45-D2650-100ML) treatments were carried out 48 h post-transfection. For such treatments, ARPE-19 cells were incubated either in media containing 20 µg/mL Tm (Tm-treated samples) or in media with 2.37 µL/mL DMSO (DMSO-treated samples) for 24 h immediately prior to sample collection. The Tm was resuspended in DMSO; therefore, the amount of DMSO is the same in both conditions. A cell death analysis was carried out to determine a sub-lethal Tm concentration and treatment duration [Supplementary Fig. S2]. For the cell death analysis, ARPE-19 cells were grown to approximately 90% confluency, treated with varying concentrations of Tm or DMSO for 24 h, and imaged on a ZOE Fluorescent Cell Imager (Bio-Rad [Hercules, CA, USA], Cat#: 1450031).

### Terminal deoxynucleotidyl transferase dUTP nick end labeling

TUNEL staining was carried out using a FITC TUNEL assay kit (Abcam, Cat#: ab66108) according to the manufacturer’s instructions with minor alterations. Briefly, ARPE-19 cells were collected 24 h after treatment with DMSO or Tm. The cells were dissociated with TrypLE Express (Fisher Scientific, Cat#: 12-605-010), collected, and fixed with 1% paraformaldehyde (Fisher Scientific, Cat#: 50-980-487, diluted in 1x phosphate-buffered saline [PBS]) for 15 min on ice. After each incubation and each wash, the cells were collected via centrifugation at 300×*g* for 2 min. The cells were then washed twice with 1x PBS, permeabilized with ice-cold 70% ethanol for 30 min, and stored at −20 °C until ready for use. On the day of the analysis, fixed cells were washed with Wash Buffer, incubated with Staining Solution, then washed with Rinse Buffer as directed by the manufacturer’s protocol; however, the cells were not stained with propidium iodide. The Wash Buffer, Staining Solution components, and Rinse Buffer were provided in the FITC TUNEL assay kit. The cells were analyzed using a BD LSRFortessa Cell analyzer (BD Biosciences [Franklin Lakes, NJ, USA]) and Floreada.io software. Regarding the analysis of the TUNEL assay, non-transfected ARPE-19 cells (neg con) served as the control for both the knockdown and overexpression samples. For each control, sample size (*N*) = 10. For each experimental condition, *N* = 5. The sample size was calculated to ensure a power of 0.8 with a type I error rate of 5%. Comparisons were made using unpaired, two-tailed *t*-tests with Welch’s correction. Sample sets were determined to be normally distributed using the Shapiro-Wilk test. Variances were calculated for each sample set, and, being found dissimilar, Welch’s correction was used. Having addressed the four assumptions of the two-sample *t*-test (independence, normality, homogeneity of variances, and random sampling), we determined that our chosen statistical analysis was justified.

### RNA isolation

APRE-19 cells were collected 48 h post-transfection. RNA was isolated using Tri-Reagent (Molecular Research Center Inc. [Cincinnati, OH, USA], Cat#: TR 118) following the manufacturer’s protocol. After the addition of the Tri-Reagent, the samples were mixed well by inversion, transferred to phase-lock heavy tubes (VWR [Radnor, PA, USA], Cat#: 10847-802), and incubated at room temperature for 5 min. Chloroform (200 µL) was added to each sample, followed by vigorous mixing for 15 s and then a 15-min incubation at room temperature. Samples were centrifuged, and the aqueous (top) phase was transferred to a new 1.5 mL microcentrifuge tube. To remove any contaminating phenol, 400 µL chloroform was added, vigorously mixed, incubated at room temperature for 2 min, and centrifuged. The aqueous phase containing the RNA was transferred to a new 1.5 mL microcentrifuge tube. Each volume of RNA solution was then thoroughly mixed with 1/10 volume of 3 M sodium acetate, 1 volume of isopropanol, and 0.5 µL RNA-grade glycogen. Samples were incubated at −80 °C for 1 h to precipitate RNA, centrifuged to pellet the RNA, and the supernatant was discarded. To wash the RNA pellets, 75% ethanol was added, then briefly vortexed and centrifuged. Ethanol was removed, and this wash was repeated. Following the second wash, ethanol was removed, and pellets were air-dried for 5–10 min. RNA was resuspended in DNase-free, RNase-free water and quantified on a Qubit 4 Fluorometer (Thermo Fisher [Waltham, MA, USA], Cat#: Q33238) using a Qubit RNA BR Assay Kit (Thermo Fisher, Cat#: Q10210). Contaminating DNA was removed via DNase I digestion, and the RNA was purified through ethanol precipitation.

### Real-time quantitative PCR

Isolated RNA served as a template for cDNA synthesis using SuperScript III First-Strand Synthesis System (Thermo Fisher, Cat#: 18080051) with oligo(dT) primers according to the manufacturer’s instructions. RT-qPCR was carried out on a CFX96 Real-Time System (Bio-Rad) attached to a C1000 Touch Thermal Cycler (Bio-Rad) using iTaq Universal SYBR Green Supermix (Bio-Rad, Cat#: 1725121) according to the manufacturer’s instructions. The primer sequences used for qPCR analysis are given in Supplementary Table S2. Regarding fold change analysis, ARPE-19 cells transfected with TYE 563 Transfection Control DsiRNA served as the control for the knockdown samples, while ARPE-19 cells transfected with GFP-expressing plasmid served as control for the overexpression samples. For each control and condition, *N* = 3.

### ELISA experimentation

ARPE-19 cells were collected 48 h post-transfection. The cells were washed once with 1x PBS and then lysed through the addition of 1x Radioimmunoprecipitation Assay (RIPA) buffer (Abcam, Cat#: ab156034). The lysates were scraped from the culture dishes, transferred to 1.5 mL microcentrifuge tubes, and rotated at 4 °C for 1 h. The lysates were then centrifuged at 15,000×*g* for 5 min at 4 °C to pellet the cell debris, and the supernatants were transferred to new 1.5 mL microcentrifuge tubes. Protein concentrations were determined using a Thermo Scientific Pierce BCA Protein Assay Kit (Fisher Scientific, Cat#: PI23227). ELISA experiments were conducted using the Human DR4 SimpleStep ELISA Kit (Abcam, Cat#: ab282881), the Human Cleaved Caspase-3 (Asp175) SimpleStep ELISA Kit (Abcam, Cat#: ab220655), and the Phospho-MLKL (S358) and Total MLKL ELISA Kit (Abcam, Cat#: ab279863) according to the manufacturer’s instructions. For the DR4 and cleaved-CASP3 detection, we used 125 ng/µL as the protein concentration for the 50 µL sample inputs, and for phos-MLKL detection, we used 250 ng/µL as the protein concentration for the 100 µL sample inputs. Endpoint readings were conducted at 450 nm and analyzed on a Synergy HT (BioTek) plate reader. Regarding fold change analysis, ARPE-19 cells transfected with TYE 563 Transfection Control DsiRNA served as the control for the knockdown samples, while ARPE-19 cells transfected with GFP-expressing plasmid served as control for the overexpression samples. For the comparison of phos-MLKL between samples, the samples were first normalized using the pan-MLKL absorbance readings (which were obtained using the pan-MLKL antibody provided in the ELISA kit). One sample (a replicate of TNFRSF10A-OE of the TNFRSF10A ELISA) failed to provide sufficient protein and was thus excluded from the analysis. For the TNFRSF10A-OE sample set of the TNFRSF10A ELISA (reported in Fig. 2C), *N* = 2. For all other samples, *N* = 3.

### RNA-fluorescent in situ hybridization

ARPE-19 cells were seeded onto 8-well chamber slides and were grown to confluence. Cells were prepared using the ViewRNA Cell Plus Assay Kit (Fisher Scientific, Cat#: 88-19000-99) according to the manufacturer’s protocol with the minor alteration of fixation and permeabilization using 3:1 methanol:glacial acetic acid for 30 min at room temperature. To stain the nuclei, the cells were incubated in Hoechst solution. The cells were then mounted and visualized using a Leica (Wetzlar, Germany) TCS SPE confocal microscope.

## Supplementary information

Supplemental Table 1

Supplemental Table 2

Supplemental Figure Captions

Supplemental Figure 1

Supplemental Figure 2

Supplemental Figure 3

## Data Availability

All data generated or analyzed during this study are included in this published article [and its supplementary information files].
