# Supplementary material for: Dysregulation of a lncRNA within the TNFRSF10A locus activates cell death pathways
Source: Cell Death Discov. 2023 Jul 13;9:242. doi: 10.1038/s41420-023-01544-5 (PMC10344863; doi:10.1038/s41420-023-01544-5)
Supplement: Supplementary file 3 — Supplemental Figure Captions [file 41420_2023_1544_MOESM3_ESM.docx]

**Supplemental Figure 1: *AC100861.1* and *TNFRSF10A* transcripts possess sequence homology.** Pairwise sequence alignment of *AC100861.1* and *TNFRSF10A* was conducted using the EMBOSS Water alignment tool, revealing 43.1% similarity. Teal regions highlight areas of homology between the *AC100861.1* and *TNFRSF10A* transcripts.

**Supplemental Figure 2: Cell death analysis of tunicamycin treatment of ARPE-19 cells.** Images of ARPE-19 cells subjected to varying amounts of tunicamycin (**A**) and DMSO (**B**) for 24 hours as indicated. Mass cell death is noticeable at the 50 µg/mL, 100 µg/mL, and 200 µg/mL concentrations of tunicamycin. Scale bar indicates 200 µm.

**Supplemental Figure 3: PKCA expression is slightly altered upon *AC100861.1* and *TNFRSF10A* dysregulation.** Graph indicates the expression level changes (compared to control) in ARPE-19 cells transfected with: siRNA targeting *AC100861.1* (AC100861.1-KD), siRNA targeting *TNFRSF10A* (TNFRSF10A-KD), *AC100861.1* overexpression vector (AC100861.1-OE), and *TNFRSF10A* overexpression vector (TNFRSF10A-OE). Expression levels of *PKCA* were measured by RT-qPCR. Gray dotted line corresponds to fold change = 1. For all samples, N = 3. Error bars indicate the standard error of mean.
