# Supplementary figures and images for: Dysregulation of a lncRNA within the TNFRSF10A locus activates cell death pathways
Source: Cell Death Discov. 2023 Jul 13;9:242. doi: 10.1038/s41420-023-01544-5 (PMC10344863; doi:10.1038/s41420-023-01544-5)

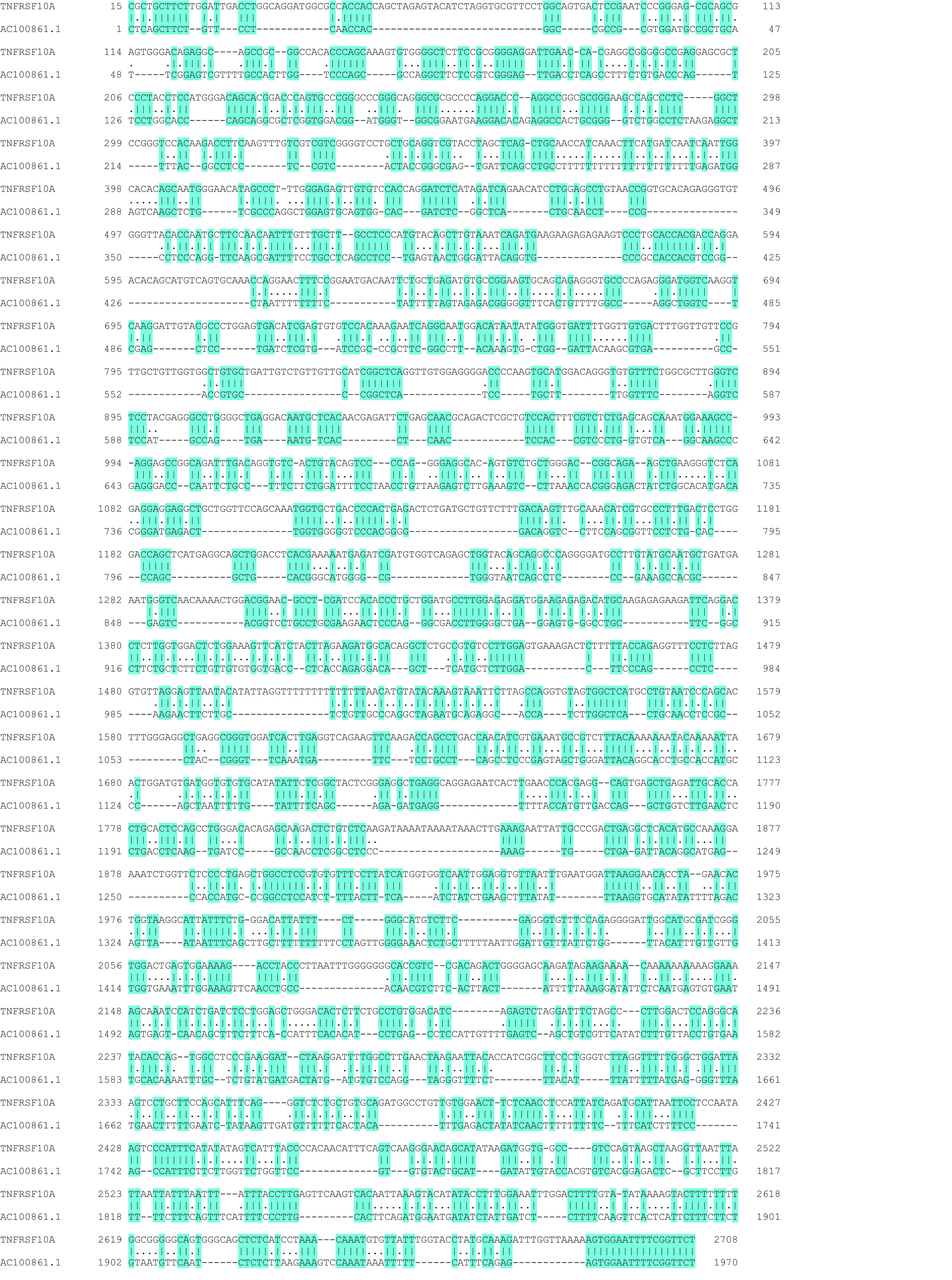

Supplement: Supplementary file 4 — Supplemental Figure 1 [file 41420_2023_1544_MOESM4_ESM.tif]

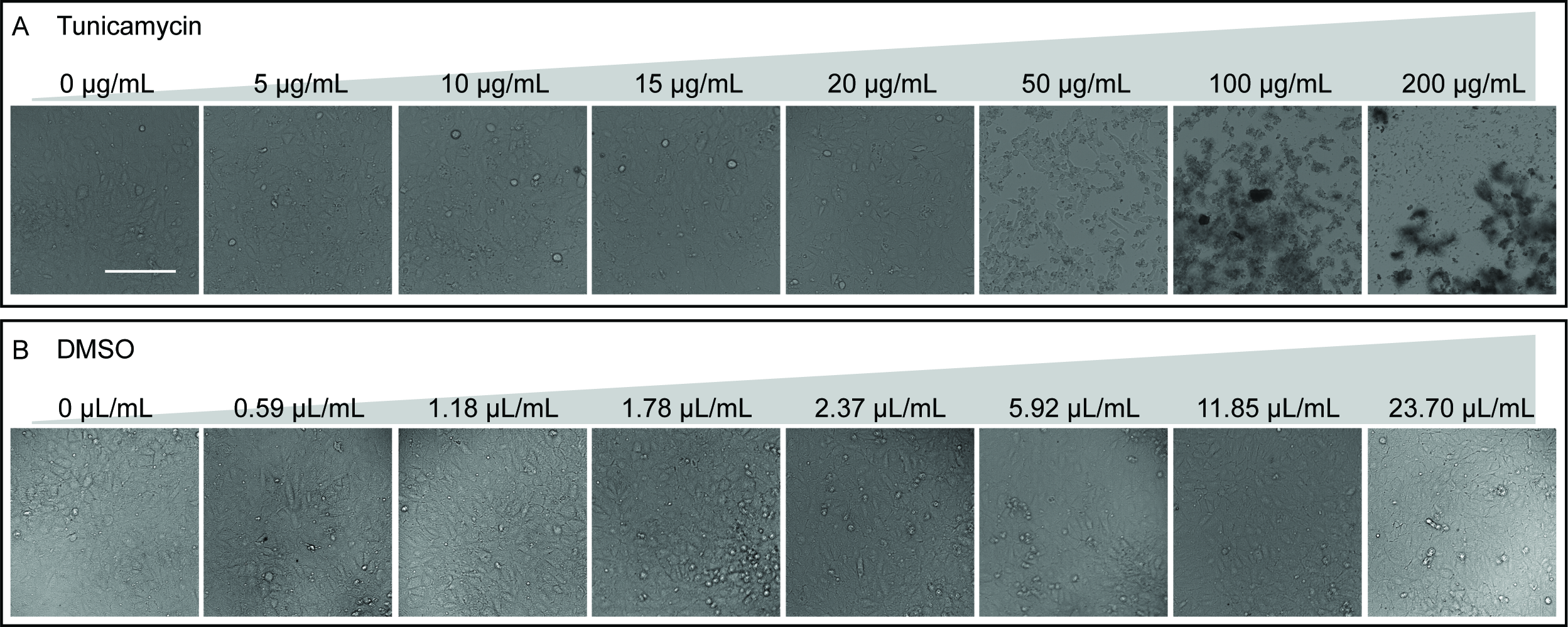

Supplement: Supplementary file 5 — Supplemental Figure 2 [file 41420_2023_1544_MOESM5_ESM.tif]

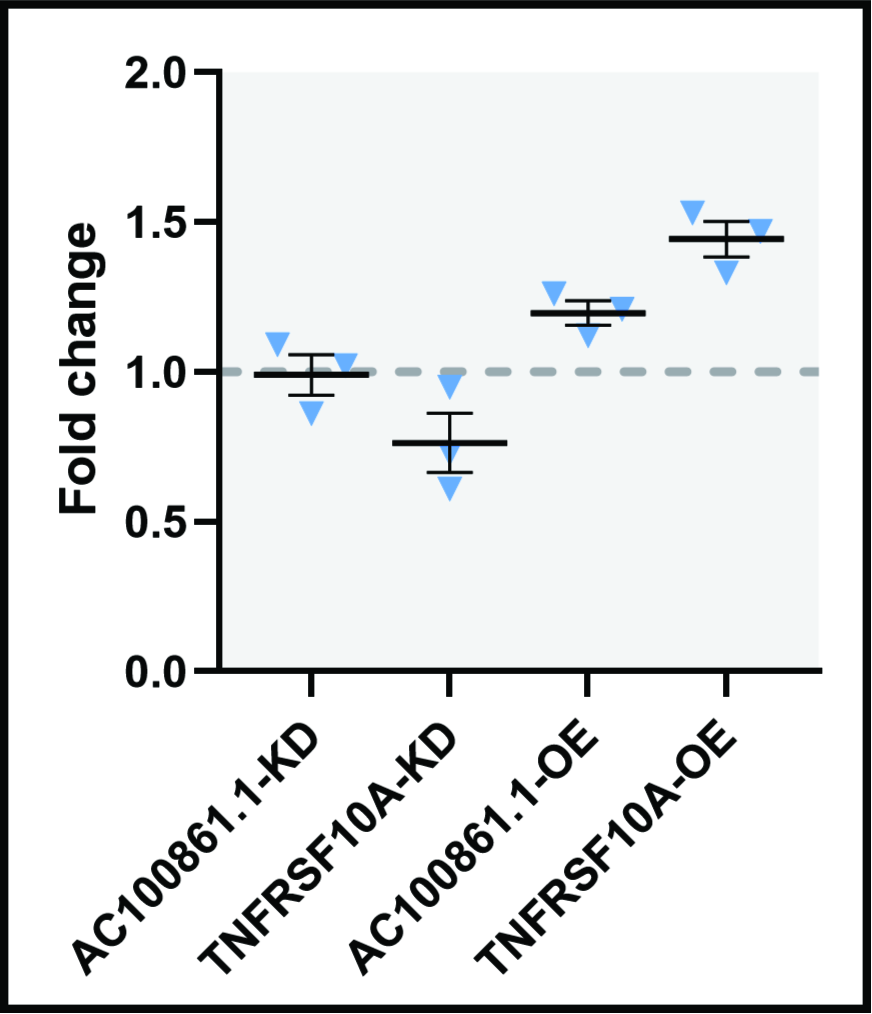

Supplement: Supplementary file 6 — Supplemental Figure 3 [file 41420_2023_1544_MOESM6_ESM.tif]
